# Plasticity of Cells and *Ex Vivo* Production of Red Blood Cells

**DOI:** 10.4061/2011/195780

**Published:** 2011-07-07

**Authors:** Takashi Hiroyama, Kenichi Miharada, Ryo Kurita, Yukio Nakamura

**Affiliations:** Cell Engineering Division, RIKEN BioResource Center, Koyadai 3-1-1, Tsukuba, Ibaraki 305-0074, Japan

## Abstract

The supply of transfusable red blood cells (RBCs) is not sufficient in many countries. If transfusable RBCs could be produced abundantly from certain resources, it would be very useful. Our group has developed a method to produce enucleated RBCs efficiently from hematopoietic stem/progenitor cells present in umbilical cord blood. More recently, it was reported that enucleated RBCs could be abundantly produced from human embryonic stem (ES) cells. The common obstacle for application of these methods is that they require very high cost to produce sufficient number of RBCs that are applicable in the clinic. If erythroid cell lines (immortalized cell lines) able to produce transfusable RBCs *ex vivo* were established, they would be valuable resources. Our group developed a robust method to obtain immortalized erythroid cell lines able to produce mature RBCs. To the best of our knowledge, this was the first paper to show the feasibility of establishing immortalized erythroid progenitor cell lines able to produce enucleated RBCs *ex vivo*. This result strongly suggests that immortalized human erythroid progenitor cell lines able to produce mature RBCs *ex vivo* can also be established.

## 1. Introduction

Transfusion therapies involving RBCs, platelets, and neutrophils depend on the donation of these cells from healthy volunteers. However, unpredictable adverse results can ensue from transfusion therapies because of the donation of cells from a very large number of anonymous volunteers. For example, transfusion of blood products that include hazardous viruses or prions is difficult to prevent completely, because, occasionally, tests to detect them yield pseudo-negative results. There is little doubt that RBCs, platelets, and neutrophils produced *ex vivo* would be candidate materials to replace cells donated from such a large group of anonymous individuals.

The development of technologies such as PCR and gene knockout that enable the manipulation of an organism's genetic material contributed tremendously to progress in the life sciences in the final decades of the last century. This century looks to continue this progress through the development of further new technologies relating to cell manipulation.

## 2. Discovery of Plasticity in Terminally Differentiated Cells

It was believed for a long time that epigenetic modifications in differentiated somatic cells were irreversible. This meant that terminally differentiated cells could never return to being immature cells. However, in 1962, it was reported that the nuclei of somatic cells of an amphibian (frog) were reprogrammed following transfer into enucleated unfertilized eggs [1]. Following transfer of a somatic cell nucleus, the egg could undergo cell division and differentiate to produce an adult frog. This result clearly indicated that epigenetic modifications in terminally differentiated somatic cells were reversible. Dr. John Gurdon, who performed this groundbreaking study, received the Albert Lasker Basic Medical Research Award in 2009.

Initially, many biologists believed that this reversibility of epigenetic modifications in terminally differentiated cells was restricted to amphibian somatic cells and did not occur in mammalian somatic cells. However, in 1997, a nuclear transfer experiment in sheep in which somatic nuclei were transferred into unfertilized eggs showed that epigenetic modifications in terminally differentiated mammalian somatic cells were also reversible [2]. This experiment famously resulted in the birth of the first live cloned sheep, named “Dolly”.

## 3. Immortalization of ES Cells

The methodology for isolating and culturing mouse ES cells was first developed in 1981 [3] and has aided research in a wide range of biological studies. Dr. Martin Evans, who developed the technology for establishing mouse ES cell lines, was awarded a Nobel Prize in 2007 together with Dr. Mario Capecchi and Dr. Oliver Smithies, who developed homologous recombination technology in mouse ES cells. As a result of these technical advances, functional analysis of genes has progressed considerably using mice with gene knockouts or other genetic modifications.

It is well known that mouse cells can be immortalized simply by continuous *in vitro* culture, for example, using the so-called “3T3 protocol”. One widely exploited example of an immortalized cell line is NIH3T3, which continues to be used in a wide range of experiments. In contrast, it is not possible to immortalize human somatic cells in a similar manner and this difficulty gave rise to the widespread assumption that it would not be possible to establish human ES cell lines. However, in 1998, 17 years after the first establishment of mouse ES cell lines, it was reported that human ES cell lines could also be produced by continuous *in vitro* culture [4].

## 4. Therapeutic Cloning

The ability to reprogram mammalian somatic cells by nuclear transfer and to establish human ES cell lines stimulated medical scientists to investigate the creation of ES cell lines using nuclear transfer as a potential means of achieving “therapeutic cloning”. If this technology could be established as a viable therapy, then patients who would benefit from somatic cell transplantation could be treated with nuclear-transferred ES cells produced using their own somatic cells, which would avoid the possibility of transplant rejection as the cells possess the same major histocompatibility (MHC) antigens as host tissue.

Although an earlier report of successful therapeutic cloning by a group in Korea proved false, it was recently reported that primate ES cell lines have been established by nuclear transfer technology [5]. Since unfertilized primate eggs are much more fragile than those of rodents, it may still take some time to establish the technology for use in human therapeutic cloning. However, the prospect of using such therapy no longer seems to be so distant.

## 5. A Search for Alternative Technologies to Therapeutic Cloning

An important limitation to the use of therapeutic cloning is that it requires unfertilized eggs. Human eggs are very difficult to obtain, and, moreover, their use for this purpose also raises serious ethical issues. For these reasons, a search has been initiated for alternative methodologies that avoid nuclear transfer. One approach has been to search for factors in unfertilized eggs that may be required for the reprogramming of transferred somatic nuclei. Another avenue of research has been to elucidate which genes specifically function in ES cells, since these genes may maintain the undifferentiated state of ES cells, and thus might be able to induce reprogramming of nuclei in terminally differentiated somatic cells.

The research group led by Dr. Shinya Yamanaka reported the first success in the latter approach. They were able to induce differentiated mouse somatic cells to become pluripotent stem cells by the application of four defined factors [6]. The enforced expression of the transcription factors Oct3/4, Sox2, Klf4, and c-Myc in terminally differentiated somatic cells induced cellular reprogramming and changed the cells into ES-like pluripotent stem cells. These reprogrammed cells were named “induced pluripotent stem (iPS) cells”. Subsequently, in the year after establishment of human iPS cell lines was first reported, several other groups also succeeded with this methodology [7–10]. Dr. Shinya Yamanaka, who developed the method, was given the Albert Lasker Basic Medical Research Award in 2009 together with Dr. John Gurdon.

The mechanisms underlying the reprogramming of terminally differentiated somatic cells following the enforced expression of the four factors remain to be elucidated. It is now known that expression of these factors after exogenous introduction is completely suppressed in established iPS cells. Thus, the factors seem to be required only for the reprogramming process but not for maintenance of pluripotency. Regardless of the mechanisms involved, this discovery clearly indicated that terminally differentiated somatic cells could be reprogrammed without nuclear transfer into unfertilized eggs and opened a new dawn for therapeutic cloning [11–13].

## 6. *Ex Vivo* RBC Production from Hematopoietic Stem/Progenitor Cells

The rapid progress relating to cell manipulation technology described above prompted many scientists in various fields to consider cell therapy using the cells produced and/or manipulated *ex vivo*. The scientists in the field of hematology are naturally aiming to produce the terminally differentiated blood cells able to use in the clinic. RBC transfusion was the first transplantation procedure to be established and is now routine and indispensable for many clinical purposes. However, in many countries, the supply of transfusable materials is not always sufficient. In Japan, for example, the supply of RBCs with an AB/RhD(−) phenotype is always lacking, because individuals with this RBC phenotype are rare. This problem of inequalities in the supply and demand for RBCs has stimulated interest in the development of *ex vivo* procedures for the generation of functional RBCs from hematopoietic stem cells or progenitor cells.

The hematopoietic stem cells that are present in bone marrow and umbilical cord blood are promising materials for *ex vivo* production of RBCs. In particular, umbilical cord blood cells are readily available, as they are usually discarded. Provided the mother of a neonate consents to use of the umbilical cord, this material can provide a useful resource without any further complicating critical or ethical concerns.

Neildez-Nguyen et al. reported that human erythroid cells (nucleated cells) produced on a large scale *ex vivo* could differentiate *in vivo* into enucleated RBCs [14]. They developed a culture protocol to expand CD34^+^ erythroid progenitor cells based on a 3-step expansion of cells by sequential supply of specific combinations of cytokines to the culture medium [14]. This study demonstrated that erythroid progenitor cells produced *ex vivo* from hematopoietic stem and/or progenitor cells could have a clinical application as an alternative method for transfusing terminally differentiated RBCs. Later, the same group described an *ex vivo* methodology for producing fully mature human RBCs from hematopoietic stem/progenitor cells [15]. The enucleated RBCs produced by this approach are potentially even more valuable, as they should be functional immediately after transfusion without requiring time for enucleation as is necessary with the erythroid cells.

## 7. Enucleation of Erythroid Progenitor Cells

The mechanism of erythroblast enucleation, a critical step in RBC production, has not yet been fully elucidated [16, 17]. The role of the interaction of erythroblasts with other cells, such as macrophages, is a controversial topic in this process [18–22]. Macrophages in retinoblastoma gene- (*Rb-*) deficient embryos are unable to physically interact with erythroblasts, and RBC production is impaired in these embryos [21]. In addition, *in vitro* production of enucleated RBCs from immature hematopoietic progenitor cells proceeds efficiently in the presence [15] but not in the absence [14] of feeder cells.

**Figure 1:**
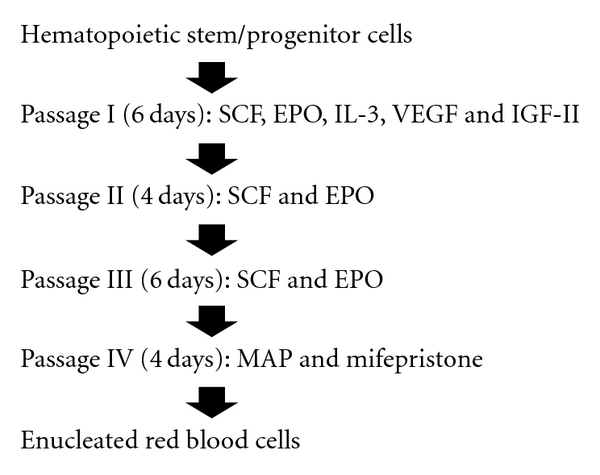
Culture protocol for the efficient production of enucleated red blood cells without feeder cells from hematopoietic stem/progenitor cells. Passage I*∼*III are the steps to expand erythroid progenitor cells. Passage IV is the step to induce enucleation of progenitor cells. SCF, stem cell factor; EPO, erythropoietin; IL-3, interleukin-3. VEGF, vascular endothelial growth factor; IGF-II, insulin-like growth factor-II; MAP, mixture of D-mannitol, adenine, and disodium hydrogen phosphate dodecahydrate.

However, enucleation can apparently be initiated *ex vivo* in erythroblasts that have been induced to differentiate *in vivo* to a developmental stage that is competent for nuclear self-extrusion [22, 23]. Consistent with these findings, our group discovered a method to produce enucleated RBCs efficiently *ex vivo* without use of feeder cells [24]. Our system for expanding erythroid progenitor cells and inducing efficient enucleation of those progenitor cells is shown in Figure 1.

The method we developed included VEGF and IGF-II in the culture medium [24]. These two factors have been reported to promote the survival, proliferation, and/or differentiation of hematopoietic progenitors [25–27]. Consistent with these findings, these factors promoted the expansion of erythroid progenitors [24]. However, a much more important feature of our culture system is that it allowed erythroid cells to differentiate to a developmental stage competent for nuclear self-extrusion [24]. It has generally been thought that efficient enucleation of erythroblasts is largely dependent on signals mediated by cells in their local environment [18–21]. However, the data we reported demonstrate that the interaction of erythroblasts with other cells is not necessary for efficient erythroblast enucleation [24]. Signals mediated by humoral factors appear to be sufficient for the efficient autonomous completion of erythroblast enucleation. In addition, since culture without the use of feeder cells is technically easier and less expensive, the method we developed has the potential to be a cost-effective means of producing transfusable RBCs on a large scale from immature hematopoietic stem/progenitor cells.

## 8. RBC Production from ES/iPS Cells

ES/iPS cells possess the potential to produce various differentiated cells able to function *in vivo,* and thus represent another promising resource for RBC production *ex vivo*. Furthermore, since ES/iPS cell lines are immortalized, they can be used repeatedly and have potential to produce abundant differentiated cells in the quantities required for clinical use. However, it will be important to carry out routing screening of the ES/iPS cell lines for *de novo* chromosomal aberrations and/or genetic mutations that may arise during culture, before these long-term cell cultures are applied in the clinic. Unsurprisingly, there is now a widespread and enthusiastic debate on standardization of the characteristics of ES/iPS cells for regenerative medicine protocols that exploit these cell lines. In our opinion, since chromosomal aberrations and genetic mutations are inevitable in long-term cell cultures, only ES/iPS cell lines that have been cultured for a limited period, for example, less than 30 passages, should be selected for clinical use.

Hematopoietic cells, including those in the erythroid lineage, have been generated from mouse ES cells [28–31], nonhuman primate ES cells [32–34], and human ES cells [35–41]. Our group has also established a long term *in vitro* method for culturing hematopoietic cells derived from ES cells of the nonhuman primate, the cynomolgus monkey [27]. Recently, abundant productions of mature RBCs from human ES cells [42] and human iPS cells [43] were also reported.

## 9. Establishment of Immortalized Erythroid Progenitor Cell Lines Able to Produce Enucleated RBCs

As described above, we can now produce mature RBCs by *in vitro* culture of ES/iPS cells or the hematopoietic stem/progenitor cells present in umbilical cord blood. In practice, however, the efficiency of RBC generation varies with the quality of the ES/iPS cell line, or the umbilical cord blood sample. Since ES/iPS cell lines can be utilized repeatedly, derivation of RBCs from ES/iPS cells appears to be more practical. However, even with optimal experimental procedures and the most appropriate ES/iPS cell line the generation of abundant RBCs directly from ES/iPS cells is a costly and time-consuming process. If immortalized human erythroid progenitor cell lines can be established that have efficient production of mature RBCs, they would provide a much more useful resource than ES/iPS cell lines.

Several mouse and human erythroid cell lines have been established. However, to the best of our knowledge, there is no cell line that can efficiently differentiate into enucleated RBCs. It is generally difficult to establish hematopoietic cell lines from adult hematopoietic stem and progenitor cells, as both are sensitive to DNA damage and are unable to maintain the lengths of telomere repeats on serial passage [43]. In contrast, ES cells are relatively resistant to DNA damage and maintain telomere lengths on serial passage [44]. Therefore, these characteristics of ES/iPS cells may be advantageous for the establishment of cell lines, since differentiated cells derived from ES/iPS cells may retain them. In fact, an erythroid cell line has been established from *in vitro*-differentiated GATA-1-deficient mouse ES cells [45].

**Figure 2:**
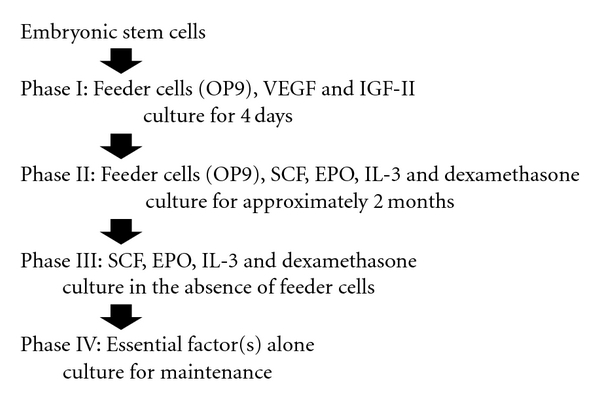
Culture protocol to establish erythroid progenitor cell lines from embryonic stem cells. In most cases, the cells failed to proliferate within two months of the initial induction of differentiation from ES cells. Induced cells that could proliferate continuously for approximately two months (60 days) were subsequently cultured in the absence of OP9 cells and in the presence of hematopoietic humoral factors. Cells that could proliferate in the absence of OP9 cells were cultured further. Approximately four months after the initial induction of differentiation of the cells, we evaluated the factors that were essential for the proliferation of each cell line. VEGF, vascular endothelial growth factor; IGF-II, insulin-like growth factor-II; SCF, stem cell factor; EPO, erythropoietin; IL-3, interleukin-3.

Recently, we developed a robust method to obtain differentiated cell lines following the induction of hematopoietic differentiation of mouse ES cells (Figure 2) and established five independent hematopoietic cell lines using this method [46]. Three of these lines exhibited characteristics of erythroid cells, and they were designated **m**ouse **E**S cell-**d**erived **e**rythroid **p**rogenitor (MEDEP) cell lines. Although their precise characteristics varied, each of the MEDEP lines could differentiate *in vitro* into more mature erythroid cells, including enucleated RBCs. Following transplantation into mice suffering from acute anemia, MEDEP cells proliferated transiently and subsequently differentiated into functional RBCs. Treated mice showed a significant amelioration of acute anemia. In addition, MEDEP cells did not form tumors following transplantation into mice. This paper was the first to demonstrate the feasibility of establishing immortalized erythroid cell lines able to produce mature RBCs.

**Figure 3:**
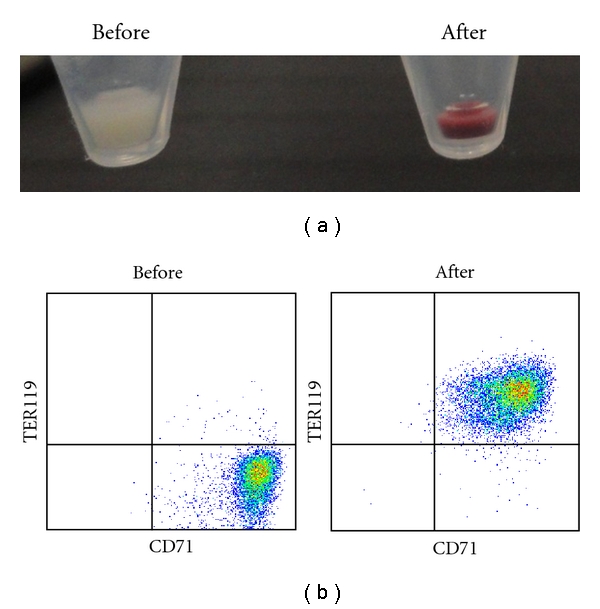
*In vitro* differentiation of a MEDEP cell line, MEDEP-BRC5. MEDEP-BRC5 cells cultured continuously for more than one and a half year was analyzed. The *in vitro *differentiation of MEDEP-BRC5 was performed by culture for four days after deprivation of stem cell factor and addition of erythropoietin. (a) Cell pellets before and after *in vitro* differentiation. Red cell pellet indicates abundant hemoglobin production in the cells. (b) Flow cytometric analyses. Before and After, the cells before and after *in vitro* differentiation; CD71, transferrin receptor; TER119, a cell surface antigen specific for mature erythroid cells.

**Figure 4:**
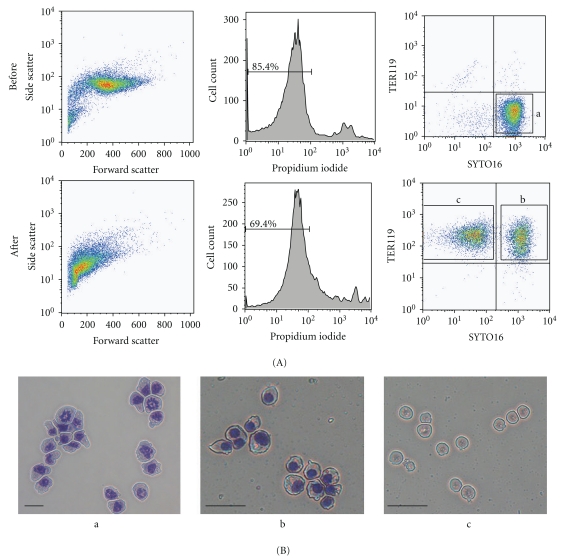
*In vitro* enucleation of a MEDEP cell line, MEDEP-BRC5. The *in vitro* differentiation of MEDEP-BRC5 was performed as described in Figure 3. (A) Flow cytometric analyses. Before and After, the cells before and after *in vitro* differentiation. Percentages of propidium iodide-negative viable cells are shown. TER119, see Figure 3. SYTO16, a cell membrane-permeable fluorochrome dye to stain nucleic acids. Following *in vitro* differentiation, 52% of the cells were TER119-positive and SYTO16-negative cells, that is, the cells lacking nuclei. (B) Morphology of cells collected from the a, b, and c fractions shown in (A). Scale bars indicate 20 *μ*m.

After the work above, we have continuously cultured the established MEDEP cell lines so as to observe whether the characteristics of them were stable. After long-term cultures for more than one and a half year, all MEDEP cell lines maintained the characteristics able to differentiate into mature erythroid cells producing hemoglobin abundantly (Figure 3). Of note, the characteristics of one of the MEDEP cell lines, MEDEP-BRC5, have changed to that able to produce enucleated RBCs very efficiently; that is, more than 50% of the cells were the enucleated RBCs following the induction of differentiation into mature erythroid cells (Figure 4). This result demonstrates that the interaction of erythroid progenitor cells with other cells is not necessary for efficient enucleation.

At present, the mechanism underlying the establishment of differentiated cell lines from ES cells has not been elucidated. Nevertheless, the data we reported clearly indicate that useful erythroid cell lines can be reproducibly obtained from mouse ES cells. Given that differentiation strategies developed for mouse ES cells often differ from those applied to human ES cells [47], the method we developed [46] may not be directly applicable to human ES cells and will require some modification.

## 10. iPS Cells as a Source for Establishing Immortalized Erythroid Progenitor Cell Lines

To establish the MEDEP cell lines, we screened eight types of mouse ES cell line and succeeded in establishing MEDEP cell lines from three of these [46]. By extrapolation from this result, it may be that many more human ES cell lines than currently available worldwide will be necessary to establish usable erythroid cell lines. In this context, the establishment of human iPS cell lines [7–10] should help to solve the problem of a potential shortfall, since human iPS cells have very similar characteristics as human ES cells.

Therefore, we attempted to establish human iPS cell lines and were able to establish a number of human iPS cell lines using fibroblast-like cells derived from neonatal tissues [48]. Fortunately, we were able to induce abundant numbers of hematopoietic cells from some of these iPS cell lines and also to establish immortalized hematopoietic cell lines from the induced hematopoietic cells (unpublished results). Currently, we are investigating the characteristics of these immortalized hematopoietic cell lines. Some seem to be erythroid cell lines.

## 11. Clinical Application of Erythroid Progenitor Cell Lines

We reported that MEDEP cells did not exhibit tumorigenicity *in vivo* [46]. Nevertheless, the tumorigenic potential of any human erythroid cell line will need to be thoroughly analyzed prior to clinical use [49, 50]. In addition, it may be advisable to engineer these cells in such a way that they are eliminated if a malignant phenotype arises for any reason [51].

Alternatively, the use of terminally differentiated cells that no longer have the capability of proliferating should allow clinical applications of ES/iPS cell derivatives without the associated risk of tumorigenicity. Thus, for example, RBCs lack nuclei following terminal differentiation and are highly unlikely to exhibit tumorigenicity *in vivo*. As such, even if the original ES/iPS cells and/or their derivatives possessed abnormal karyotypes and/or genetic mutations, they might, nonetheless, be useful for clinical applications, provided that they can produce enucleated RBCs. Indeed, the MEDEP lines included many cells possessing abnormal karyotypes; however, the vast majority of the cells in each cell line, nevertheless, differentiated into mature erythroid cells and transplantation of these cells significantly ameliorated anemia [46].

As described in this paper, various methods have been developed that enable the *ex vivo* production of enucleated RBCs from human hematopoietic stem/progenitor cells [14, 15, 24] and ES/iPS cells [42, 43]. Therefore, once appropriate erythroid progenitor cell lines have been established, it should be possible to apply these methods for producing enucleated RBCs *ex vivo*. Since RBCs are much smaller than normal nucleated cells, RBCs produced *ex vivo* could be selected by size prior to use in the clinic so as to exclude nucleated cells, for example, by filtration. In addition, X-ray irradiation might be useful for eradicating any contaminating nucleated cells without affecting the RBCs.

Another potential obstacle to the clinical use of ES/iPS cell derivatives is that of immunogenicity [52, 53]. Transplanted MEDEP cells could not ameliorate acute anemia in mouse strains other than those from which each individual cell line was derived or in immunodeficient mice [46], suggesting immunological rejection in heterologous strains. Hence, the clinical application of erythroid cell lines will require use of many cell lines that express different major histocompatibility (MHC) antigens. However, *ex vivo*-generated RBCs need to be compatible with ABO and RhD antigens alone. Furthermore, the establishment of an immortalized human erythroid cell line lacking the genes to produce A, B, and RhD antigens would be a very useful resource for clinical application, since such a cell line would produce O/RhD(−) RBCs, which would, in theory, be transfusable into all individuals.

## 12. Conclusions

It is now highly likely that immortalized human erythroid progenitor cell lines able to produce enucleated RBCs can be established in the near future. We believe that the transfusion of RBCs produced *ex vivo* from such cell lines will become a standard procedure in the clinic.
